# Assessment of risk factors for cerebral oxygen desaturation during neonatal and infant general anesthesia: an observational, prospective study

**DOI:** 10.1186/s12871-016-0274-2

**Published:** 2016-10-28

**Authors:** Ilona Razlevice, Danguole C. Rugyte, Loreta Strumylaite, Andrius Macas

**Affiliations:** 1 Department of Anesthesiology, Lithuanian University of Health Sciences, Eiveniu str. 2, Kaunas, LT-50009 Lithuania; 2 Neuroscience Institute, Lithuanian University of Health Sciences, Kaunas, Lithuania

**Keywords:** Near-infrared spectroscopy, Neonates, Anesthesia, Cerebral oxygenation

## Abstract

**Background:**

Cerebral oxygen saturation (rSO_2_c) decrease from baseline greater than 20 % during infant cardiac surgery was associated with postoperative neurologic changes and neurodevelopmental impairment at 1 year of age. So far, there is no sufficient evidence to support the routine monitoring of rSO_2_c during general surgical procedures in children. We aimed to find out the frequency of cerebral desaturation 20 % or more from baseline and to identify possible predictors of change in cerebral oxygen saturation during neonatal and infant general surgery.

**Methods:**

Forty-four infants up to 3 months of age were recruited. Before induction of anesthesia, two pediatric cerebral sensors were placed bilaterally to the forehead region and monitoring of regional cerebral saturation of oxygen was started and continued throughout the surgery. Simultaneously, mean arterial blood pressure (MAP), pulse oximetry (SpO_2_), heart rate (HR), endtidal CO_2_, expired fraction of sevoflurane and rectal temperature were recorded. The main outcome measure was rSO_2_c value drop-off ≥20 % from baseline. Mann-Whitney *U-*test, chi-squared test, simple and multiple linear regression models were used for statistical analysis.

**Results:**

Forty-three infants were analyzed. Drop-off ≥20 % in rSO_2_c from baseline occurred in 8 (18.6 %) patients. There were no differences in basal rSO_2_c, SpO_2_, HR, endtidal CO_2_, expired fraction of sevoflurane and rectal temperature between patients with and without desaturation 20 % or more from baseline. But the two groups differed with regard to gestation, preoperative mechanical ventilation and the use of vasoactive medications and red blood cell transfusions during surgery. Simple linear regression model showed, that gestation, age, preoperative mechanical ventilation and mean arterial pressure corresponding to minimal rSO_2_c value during anesthesia (MAP_minrSO2c_) were associated with a change in rSO_2_c values. Multiple regression model including all above mentioned variables, revealed that only MAP_minrSO2c_ was predictive for a change in rSO_2_c values (β (95 % confidence interval) -0.28 (−0.52–(−0.04)) *p* = 0.02).

**Conclusions:**

Cerebral oxygen desaturation ≥20 % from baseline occurred in almost one fifth of patients. Although different perioperative factors can predispose to cerebral oxygenation changes, arterial blood pressure seems to be the most important. Gestation as another possible risk factor needs further investigation.

**Trial registration:**

The international registration number NCT02423369. Retrospectively registered on April 2015.

**Electronic supplementary material:**

The online version of this article (doi:10.1186/s12871-016-0274-2) contains supplementary material, which is available to authorized users.

## Background

Due to improved medical care many seriously ill neonates and infants survive. However, adverse neurodevelopmental outcome of survived infants still exists [1], and the etiology of cerebral lesions is not sufficiently clear [2]. Infant’s brain is vulnerable to poor blood flow and oxygenation changes, as a result long-term outcome can be affected [1].

The assessment of adequate cerebral perfusion in small children is commonly based on routine clinical parameters, whereas, invasive measurement techniques requiring central venous and/or arterial catheter access, such as jugular bulb oxygen saturation or central venous saturation are quite risky [3]. Several studies suggest using cerebral near infrared spectroscopy (NIRS) at the bedside to identify possible risk factors of adverse neurologic events. NIRS measures the regional tissue oxygen saturation of various organs and provides a reflection of the balance between tissue oxygen supply and demand. The main purpose of NIRS is to evaluate tissue perfusion and oxygenation continuously and non-invasively [3]. In 1985, Brazy and Lewis reported the first pediatric application of cerebral oxygenation monitoring in sick preterm infants [3, 4]. In 2011, Kasman N and Brady K in a review article summarized the existing evidence relating cerebral desaturation events to neurological outcome in children who had undergone cardiac surgery [5]. A few recent studies addressed the issue of adequate blood pressure based on cerebral oxygenation monitoring in children under inhaled general anesthesia with sevoflurane [6, 7]. But, so far, there is no sufficient evidence to support the routine use of NIRS during general surgical procedures in children.

Perioperative period in newborns and infants carries the risk of cerebral perfusion disturbances due to potential hemodynamic or metabolic derangements as a consequence of patient, surgery and anesthesia-related factors [8]. Furthermore, there is still lack of evidence on safe blood pressure limits with regard to cerebral blood flow autoregulation in neonates and especially premature neonates and infants [9]. In spite of that, during general surgery the central nervous system is seldom directly monitored. Previous studies which were performed in intensive care unit and during infant cardiac surgery report cerebral desaturation greater than 20 % from baseline or an absolute decrease below 50 % to be associated with postoperative neurologic changes and neurodevelopmental impairment at 1 year of age [10, 11]. Therefore the aim of the present study was to find out the frequency of cerebral desaturation events and to identify possible predictors of change in cerebral oxygen saturation during neonatal and infant general surgery.

### Methods

This prospective observational study was performed in Lithuanian University of Health Sciences, Kaunas Clinics, the department of Anesthesiology from 2013 May to 2015 November. Ethics approval was obtained from the Local Ethics Committee (Kaunas Regional Biomedical Research Ethics Committee, ref. n. BE-2-43). The patients described in this study constitute the part of the patients of the internationally registered study no NCT02423369. Informed written parental consent was obtained before enrollment of every patient.

We recruited 44 term and preterm infants younger than 3 months old, undergoing general, thoracic or urologic surgery for congenital anomalies or disease. Exclusion criteria were: cardiac surgery, any evidence of neurosurgical disease, sepsis, renal or hepatic insufficiency, hyperbilirubinemia and physical status of the patients corresponding to American Society of Anesthesiologists (ASA) classification 5^th^ class. Patients were fasted as indicated by the surgical condition or according to ASA fasting recommendations appropriate for age. After arrival to the operating room, continuous electrocardiography, pulse oximetry (SpO_2_), non-invasive blood pressure monitoring was started before anesthesia as per standard of care. All patients underwent general anesthesia with tracheal intubation and controlled ventilation. Anesthesia was induced with an inspired fraction of sevoflurane up to maximum 6 in 50 % O_2_/air. Muscle relaxants were administered to facilitate orotracheal intubation (7 patients were on positive pressure ventilation before surgery). Anesthesia was maintained with sevoflurane up to 1 minimal alveolar concentration, additional doses of fentanyl (1 mcg∙kg^−1^) and muscle relaxant. During anesthesia positive pressure controlled ventilation using circle breathing system was performed to maintain endtidal CO_2_ (etCO_2_) between 35 and 45 mmHg, whenever possible; positive end-expiratory pressure of 4 mmHg was used in all patients. Intraoperative infusion therapy was given as per standard of care and consisted of 5 % glucose 4 ml∙kg^−1^∙hour^−1^ and isotonic crystalloid 6–20 ml∙kg^−1^∙hour^−1^. According to the decision of responsible anesthesiologist hemodynamic support with additional boluses of isotonic crystalloid 10 ml∙kg^−1^ and/or vasoactive medications were administered based on assumption that hypotension was defined as mean arterial blood pressure (MAP) less than gestational age in weeks for premature newborns and less than 38 mmHg for term newborns and infants [8, 12]. Blood products were administered as indicated according to local clinical protocols, based on current recommendations.

A near-infrared spectrometer (INVOS®, SOMANETICS) was used for measurement of regional cerebral saturation of oxygen (rSO_2_c). Before induction of anesthesia, two pediatric cerebral sensors were placed bilaterally to the forehead region and rSO_2_c monitoring started. Data were captured with a sampling interval of 5 s and when stable for a period of 1–2 min, baseline value was noted. Throughout the surgery rSO_2_c monitoring was used continuously with information recorded every 5 min. Simultaneously, MAP, HR, SpO_2_, etCO_2_, expired fraction of sevoflurane and rectal temperature were recorded. As the rSO_2_c monitoring is not a standard care in our unit, clinical decisions during anesthesia were not based on rSO_2_c readings. After surgery, NIRS sensors were removed, and all patients were transferred to the intensive care unit for artificial lung ventilation, sedation and analgesia.

### Statistical analysis

Mean rSO_2_c value of the right and left electrode was calculated at every 5 min point for each patient. The lowest (minimal) rSO_2_c value of every patient, when SpO_2_ was ≥90 % for premature newborns and ≥94 % for term newborns and infants [13] was compared to baseline value (% change). The main outcome measure was rSO_2_c value drop-off 20 % or more from baseline [10]. Patients with at least one rSO_2_c value drop-off 20 % or more from baseline formed desaturation group. The normal group was formed from the patients without this criterion. Repeated measures analysis of variance (ANOVA) was used to compare normally distributed (Kolmogorov-Smirnov test) rSO_2_c values during anesthesia in normal and desaturation groups.

Mean intraoperative value of MAP, HR, SpO_2_, etCO_2_, expired fraction of sevoflurane and rectal temperature was calculated for each patient.

As some demographic, preanesthetic and anesthetic characteristics of patients were distributed abnormally nonparametric statistics was used. Continuous variables were summarized using median (min and max values) and compared between the groups (normal and desaturation) using Mann-Whitney *U-*test. Categorical variables were summarized using frequencies and percentages (%) and compared between the groups using chi-squared test. Spearman correlation coefficient was calculated to demonstrate the relationship between % change in cerebral oxygen saturation and MAP corresponding to minimal rSO_2_c value during anesthesia (MAP_minrSO2c_). Parametric *t*-test, was used to compare normally distributed (Kolmogorov-Smirnov test) cerebral oxygenation values corresponding to MAP values during anesthesia.

Patient demographic characteristics (variables) (gestation, age), preoperative mechanical ventilation and MAP_minrSO2c_ were included into a simple linear regression model as predictive factors for a % change in cerebral oxygen saturation during surgery. As all included parameters were significant for a % change in cerebral oxygen saturation, they all were included into multiple linear regression model. Regression coefficients β and 95 % confidence intervals were calculated.

The level of statistical significance was set at 0.05. All statistical tests were two-sided. The statistical analysis was performed by using IBM SPSS statistical software (SPSS v.20 for Windows).

## Results

Forty-four patients were enrolled in this study. One patient was excluded due to the low mean perioperative SpO_2_ (<90 %), thus 43 (14 preterm and 29 term) infants were analyzed. Demographic and clinical characteristics of the patients are shown in Table 1.

**Table 1:** Demographic characteristics of included patients. Data are shown as median (min-max) or proportions (n (%))

Variable	*n* = 43
Neonates 0–28 days (n (%))	36 (83.7 %)
Term (n)	26
Preterm (n)	10
Infants 29–70 days (n (%))	7 (16.3 %)
Term (n)	3
Preterm (n)	4
Infants operated in the preterm age (n (%))	–
Gestational age (weeks)	38 (25–41)
Weight (kg)	3.4 (0.8–5.0)
Age at surgery (days)	6 (0–70)
Gender	
Male (n (%))	25 (58.1 %)
Female (n (%))	18 (41.9 %)
Type of surgery	
Thoracic (n (%))	2 (4.6 %)
Abdominal (n (%))	31 (72.1 %)
Urologic (n (%))	3 (7.0 %)
Other (n (%))	7 (16.3 %)
Duration of anesthesia (min)	80 (30–260)

Median (range) % change in rSO_2_c from baseline during surgery was (−12.1) (+12.7–(−36.6)) %. Drop-off 20 % or more from baseline occurred in 8 (18.6 %) patients (desaturation group). In desaturation group absolute minimal rSO_2_c value was 66 % (41.5–71 %), whereas in normal group was 76.5 % (60.5–90 %); (*p* = 0.0004). In desaturation group duration of desaturation ranged from 5 to 90 min. Absolute minimal rSO_2_c value below 50 % for a period of 50 min was observed in one infant, who had basal rSO_2_c of 65.5 %. With regard to % change from baseline in normal (mean 9.27 %) and desaturation (mean 26.15 %) groups, and combined standard deviation 9.9 %, statistical power of our study was calculated to be 0.989.

**Fig. 1:**
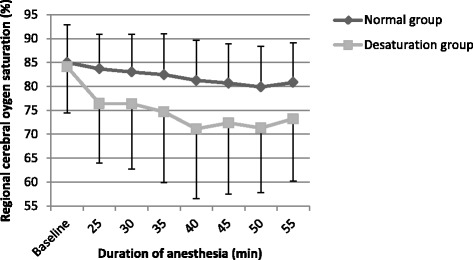
Regional cerebral oxygen saturation during anesthesia in normal and desaturation groups (data is shown as mean ± SD). The start of desaturation was observed from the 25^th^ min. Time spam of 55 min includes all patients (8) from desaturation group and 30 patients from normal group (5 patients were excluded by ANOVA because of duration of anesthesia shorter than 55 min). There was a significant difference between normal and desaturation groups (*p* = 0.041, ANOVA)

Intraoperative rSO_2_c values during anesthesia in normal and desaturation groups are shown in Fig. 1. Cerebral oxygenation values in desaturation group were lower compared to normal group (*p* = 0.041) by ANOVA).

Comparison of demographic and clinical characteristics between infants with and without cerebral oxygen desaturation events is shown in Table 2. Baseline rSO_2_c values and the majority of anesthetic parameters were not different between the groups compared. The groups differed with regard to gestation, weight, preoperative mechanical ventilation and intraoperative use of vasoactive drugs and red blood cell transfusions.

**Table 2:** Comparison of demographic and clinical characteristics between infants with and without cerebral oxygen desaturation (rSO_2_c) events 20 % or more from baseline during surgery. Data are shown as median (min-max) or proportions (n (%))

Demographic and preanesthetic parameters	Normal group (*n* = 35)	Desaturation group (*n* = 8)	*p*
Age (days)	8 (0–70)	2 (0–39)	0.09
Gestation (weeks)	38 (33–41)	35.5 (25–38)	0.007
Weight (kg)	3.4 (1.8–5.0)	2.81 (0.8–3.8)	0.04
Preoperative MAP^a^ (mmHg)	52 (36–84)	44 (33–64)	0.2
Hypotension^b^ (n (%))	2 (5.7 %)	0 (0 %)	0.5
Preoperative hemoglobin (g∙l^−1^)	165 (82–231)	147.5 (98–195)	0.13
Preoperative blood lactate (mmol/l)	2.1 (0.9–5.8)	2.3 (1.7–4.3)	0.27
Preoperative mechanical ventilation (n (%))	2 (5.7 %)	5 (62.5 %)	0.000
Baseline rSO_2_c (%)	83.5 (70–95)	87.5 (65.5-95)	0.8
Anesthetic parameters^c^			
Intraoperative SpO_2_ (%)	97.5 (91.1–99.4)	96.6 (90.5–99.9)	0.4
Intraoperative heart rate (beats per min)	143 (115.6–166.9)	149.7 (129.2–164.7)	0.4
Expired fraction of sevoflurane (%)	1.85 (0.72–2.7)	1.79 (0.5–2.6)	0.3
etCO_2_ during anesthesia (mmHg)	34.9 (20.9–50.7)	35.1 (21.1–49)	0.9
Intraoperative rectal temperature (°C)	37 (36.1–38.2)	36.9 (36–37.5)	0.2
Intraoperative MAP (mmHg)	50.0 (25.1–62.1)	39.4 (30.3–57.2)	0.06
MAP_minrSO2c_ (mmHg)^d^	47 (22–76)	43 (23–56)	0.16
Vasoactive agents (n (%))	4 (11.3 %)	4 (50 %)	0.01
Red blood cell transfusion (n (%))	2 (5.7 %)	4 (50 %)	0.001
Postanesthesia parameters			
Postoperative blood lactate level (mmol/l)	1.45 (1.1–2.2)	1.5 (1.0–3.0)	0.83
Hemoglobin change during surgery (g∙l^−1^)	−21 ((−44)–18)	−35 ((−43)–23)	0.16

^a^mean arterial blood pressure

^b^hypotension was defined as MAP less than gestational age in weeks for premature newborns and less than 38 mmHg for term newborns and infants [8, 12]

^c^average intraoperative value of heart rate, SpO_2_, etCO_2_, expired fraction of sevoflurane, rectal temperature, MAP was calculated for each patient

^d^mean arterial blood pressure corresponding to minimal rSO_2_c during anesthesia

**Fig. 2:**
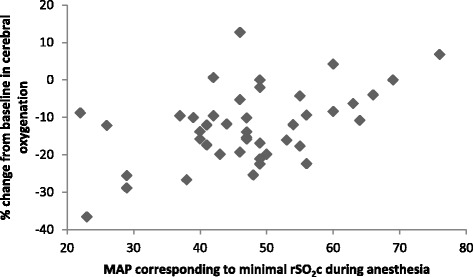
A relation between % change in cerebral oxygenation (rSO_2_c) and mean arterial blood pressure (MAP) corresponding to minimal rSO_2_c during anesthesia; Spearman’s correlation coefficient, *r* = 0.37, (*p* = 0.02)

**Fig. 3:**
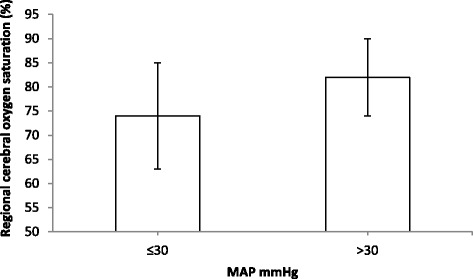
Cerebral oxygen saturation values corresponding to mean arterial blood pressure (MAP) intervals (>30 mmHg and ≤30 mmHg) during surgery. Data are shown as mean ± SD. *P* = 0.015, mean difference −7.2 (95 % CI: −12.9–(−1.4))

Simple linear regressions were calculated for demographic parameters and parameters, which were different between normal and desaturation groups (gestation, age, preoperative mechanical ventilation). The use of intraoperative vasoactive agents and red blood cell transfusions were not included because were applied in response to reduced circulation. Instead, MAP_minrSO2c_ was included into simple linear regression model as a predictor. Simple linear regression model showed, that all included predictors were associated with a % change (decrease) in rSO_2_c values during surgery compared to baseline (Table 3, (β^a^)). However, multiple regression model revealed that only MAP_minrSO2c_ was predictive for a % change (decrease) in rSO_2_c values (Table 3, (β^b^)). Relationship between % change in rSO_2_c values during surgery and MAP_minrSO2c_ is shown in Fig. 2. In addition, cerebral oxygenation values corresponding to simultaneously recorded MAP during surgery revealed that when MAP >30 mmHg, rSO_2_c values were higher than rSO_2_c values when MAP ≤30 mmHg (*p* = 0.015, mean difference −7.2 (95 % CI: −12.9–(−1.4)) (Fig. 3)).

**Table 3:** Linear regression models (simple and multiple) for % change (decrease) from baseline in cerebral oxygenation during surgery and different factors

Factor	Simple β^a^ (95 % confidence interval)	*p*	Multiple β^b^ (95 % confidence interval)	*p*
Gestation (weeks)	−1.18 (−2.22–(−0.15))	0.03	−0.54 (−1.73-0.64)	0.36
Age (days)	−0.18 (−0.36–(−0.01))	0.04	−0.22 (−0.45–0.01)	0.07
Preoperative mechanical ventilation (yes, no)	−8.78 (−16.67–(−0.09))	0.03	−2.16 (−10.53–6.22)	0.60
MAP_minrSO2c_ (mmHg)	−0.41 (−0.65–(−0.18))	0.001	−0.28 (−0.52–(−0.04))	0.02

β^a^:regression coefficient (simple linear regression model) for % change (decrease) from baseline in cerebral oxygenation during surgery

β^b^:regression coefficient (multiple linear regression model) for % change (decrease) from baseline in cerebral oxygenation during surgery

On the in-hospital follow-up which ranged from 14 days until 6 months decline in neurological function (according to the last documented clinical evaluation by pediatric neurologist) was observed in 3 patients in desaturation group, compared to none in normal group (*p* = 0.000). Two of these patients were premature, all three had undergone cardiorespiratory resuscitation after birth. Two patients from normal group subsequently died due to unfavorable course of surgical disease.

## Discussion

The main finding of a present study is that cerebral desaturation 20 % and more, compared to baseline occurred in almost one fifth of the patients during general surgery in newborns and infants. We chose a threshold of 20 % based on previous studies in cardiac and intensive care patients. Reduction from baseline greater than 20 % during surgery or in the neonatal intensive care unit was associated with postoperative neurologic changes and neurodevelopmental impairment at 1 year of age [10, 14].

Infants and especially neonates undergoing surgery represent a complex medical condition, thus not a single, but multiple patient, disease and treatment related factors can influence cerebral blood flow and cerebral oxygenation [7, 11, 15]. Although we found that gestation, age and preoperative mechanical ventilation were associated with desaturation, multiple linear regression model revealed that only arterial blood pressure was predictive of cerebral oxygen saturation decrease from baseline. So far, there is no clear evidence regarding association between hypotension and decreased cerebral oxygenation. C. Binder-Heschl and coworkers revealed that mild and short-term hypotension episodes did not influence rSO_2_c in preterm newborns [15]. Nonetheless, a recent study described positive association of intraoperative blood pressure and rSO_2_c values in a cohort of anesthetized infants up to 3 months of age [11]. In anesthetized patients, O. Rhondali et al. found that the higher the absolute MAP was during anesthesia, the higher the rSO_2_c was [6, 7].

The great majority of our patients were term and preterm neonates under 28 days of life. Appropriate blood pressure in neonates still remains a question for debate. We observed that NIRS values were lower, when MAP was 30 mmHg or less, compared to higher MAP values (Fig. 3). Safe lower limit of mean arterial blood pressure in newborn is believed to be not below the gestational age in weeks [9, 12]. Some studies report that lower limit of MAP to maintain cerebral autoregulation could be 28–30 mmHg [12]. There are findings that in preterm newborns MAP less than 30 mmHg can cause cerebral lesions which are detected by neurosonography [16]. Other studies have reported a strong association between MAP less than 30 mmHg and bad neurological outcome in preterm infants [2, 12, 16, 17]. In our study, we observed one day old premature infant who had rSO_2_c below 50 % (maximal decrease from baseline 37 %) for a period of 50 min and a simultaneous hypotension (including MAP values below 30 mmHg) for a period of 80 min. In the early postoperative period subependymal hemorrhages were noticed bilaterally, but, improving over time. Long term outcome, though, of this patient is still unknown.

During neonatal and infant anesthesia important issue is appropriate pulmonary ventilation as cerebral blood flow is affected by blood PaCO_2_ and O_2_ levels [18]. We aimed to maintain etCO_2_ within 35–45 mmHg whenever possible, however lower values were observed, which may indicate periods of hyperventilation or diminished cardiac output and consequently reduced cerebral blood flow. In spite of that, etCO_2_ during surgery did not differ between our patients with and without desaturation. However, a part of our studied patients were on mechanical ventilation preoperatively. Reasons for preoperative mechanical ventilation were: inadequate spontaneous ventilation just after delivery (*n* = 4), respiratory insufficiency due to increased intraabdominal pressure (*n* = 2), obstruction of the upper airway by tumor (teratoma) (*n* = 1). This might be a possible cause for bias as low rSO_2_c values could be linked with artificial ventilation especially when airway pressures are high, as was demonstrated in infants with respiratory distress syndrome [19–21]. Preoperative mechanical ventilation was associated with prematurity (35.7 % in preterm vs. 6.9 % in term infants) and might have reflected more difficult clinical condition of these patients. In conjunction with other factors like anemia and hypotension, prematurity can be an important factor for a decrease in cerebral oxygenation during surgery, as gestational age was associated with intraoperative cerebral oxygenation changes by simple regression model in a present study. Further studies including larger premature patient population are required to clarify this issue.

Important finding is that there were differences in anesthesia management between patients with and without cerebral oxygen desaturation 20 % and more from baseline. More patients in desaturation group received vasoactive agents and red blood cell transfusions compared to normal group patients. As the rSO_2_c monitoring is not a standard care in our unit, clinical decisions during anesthesia were not based on rSO_2_c readings. However, cerebral oxygenation monitoring was not blinded, therefore we cannot exclude that it might have influenced anesthesia management in certain cases.

The major limitation of our study is that our patient population was not homogenous. Although all patients were hemodynamically stable before surgery, they were different regarding gestation and age, and birth history, the known risk factor of cerebral lesions in infants [22]. Thus, neurological outcomes cannot be evaluated based on this study since, neurological impairment observed in 3 patients in desaturation group was not likely due to intraoperative events. Other sources of bias are: 1. Measurement of etCO_2_ in small children is not accurate. Blood gas analyses to assess PaCO_2_ would have been more precise. However, due to the ease of application and non-invasive character etCO_2_ is a part of standard monitoring during anesthesia. 2. We recorded data every 5 min, although rSO_2_c values were captured every 5 s. Therefore, we might have missed short desaturation episodes during anesthesia. 3. We performed rSO_2_c monitoring only in the forehead region and did not register oxygenation of other tissues, thus we could not compare overall tissue oxygenation to cerebral tissue oxygenation. However, blood lactate concentration did not differ between patients in normal and desaturation groups pre- and post-operatively, implying that overall perfusion was similar in both groups.

## Conclusions

Cerebral desaturation ≥20 % from baseline may occur in almost one fifth of patients. Recognition and avoidance of risk factors for potential decrease in cerebral oxygenation is the crucial task for every anesthesiologist. Although different perioperative factors can predispose to cerebral oxygenation changes, arterial blood pressure seems to be the most important. We were not able to provide strong evidence, but prematurity, as another possible risk factor cannot be ignored. Therefore, further studies are required to clarify this issue and to link cerebral desaturation episodes to patient outcome.

## Additional file

Additional file 1: “Neonatal cerebral oxygenation changes” This file contains data about demographic, clinical characteristics and cerebral oxygenation changes during anesthesia of included patients. (XLSX 37 kb)

## Abbreviations

ASA: American Society of Anesthesiologists
etCO_2_: Endtidal CO_2_
HR: Heart rate
MAP: Mean arterial pressure
MAP_minrSO2c_: MAP corresponding to minimal rSO_2_c value during anesthesia
NIRS: Near infrared spectroscopy
rSO_2_c: Regional cerebral saturation of oxygen
SpO_2_: Pulse oximetry
